# Depressive Symptoms and Addictive Behaviors in Young Adults After Childhood Trauma: The Mediating Role of Personality Organization and Despair

**DOI:** 10.3389/fpsyt.2018.00318

**Published:** 2018-07-16

**Authors:** Jürgen Fuchshuber, Michaela Hiebler-Ragger, Adelheid Kresse, Hans-Peter Kapfhammer, Human F. Unterrainer

**Affiliations:** ^1^Center for Integrative Addiction Research, Grüner Kreis Society, Vienna, Austria; ^2^University Clinic for Psychiatry and Psychotherapeutic Medicine, Medical University Graz, Graz, Austria; ^3^Institute for Pathophysiology and Immunology, Medical University Graz, Graz, Austria; ^4^Department of Religious Studies, University of Vienna, Vienna, Austria

**Keywords:** substance use disorder, depression, structural equation modeling, childhood trauma, primary emotions, personality organization, mediation

## Abstract

**Background:** There is substantial evidence that traumatic experiences in childhood increase the likelihood of mood pathology and addictive behaviors in adolescence and young adulthood. Furthermore, both forms of psychopathology have been linked to deficiencies in personality organization and a common primary emotion core. In this study, we intended to further investigate these interactions by assuming a mediating role of personality organization and despair regarding the relationship between childhood trauma and psychiatric symptom burden later in life.

**Methods:** A total sample of 500 young adults (Age: *M* = 26; *SD* = 5.51; 63.2% female) were investigated. Structural Equation Modeling was applied in order to investigate the pathways between the latent variables Childhood Trauma, Structural Deficit, Despair (comprised of the primary emotions SEEKING and SADNESS), as well as symptoms of addiction and depression.

**Results:** The results indicate that the influence of Childhood Trauma on Addictive Behaviors was mediated by Structural Deficit (*p* < 0.01), whereas its influence on Depressive Symptoms was mediated by Despair (decreased SEEKING and increased SADNESS) (*p* < 0.01). Furthermore, Addictive Behaviors seemed to be stronger represented in males (*p* < 0.001). The final model was able to explain 39% of the variance of Addictive Behaviors and 85% of the variance of Depressive Symptoms.

**Discussion:** The findings underline the importance of early experiences in the development of adult affective and personality functioning, which is linked to the development of psychiatric disorders. Regarding clinical practice, addiction treatment might focus on the improvement of personality organization, while treatment of depressed patients should primarily emphasize the restructuring of dysfunctional primary emotion dispositions.

## Introduction

The experience of childhood trauma has been discussed as a strong risk-factor for later psychopathology since the end of the Nineteenth century (1, 2). Therefore, trauma is regarded as an event so intense that it is impossible for the subject to integrate this experience on a symbolic level and thus fosters a pathological active formation of personality structure and affective forces (3). The assumption of a substantial correlation between childhood trauma and psychopathology is supported by a large number of empirical observations (4–7). More specifically, patients with a substance use disorder, as well as patients suffering from depression, frequently report histories of severe childhood maltreatment (8–10). The pathogenic effect of childhood trauma is neurobiologically explained by the development of a hyperactivity of the corticotrophin-releasing factor systems and unfavorable alterations in other stress modulating and conflict integrating systems (11, 12). On a behavioral level, this adverse influence on brain development is reflected by impaired emotional regulation which was found to mediate the relationship between childhood trauma and adult psychopathology (13, 14). Furthermore, recent studies by Schimmenti (15) and Granieri et al. (16) observed that the link between childhood trauma, adult life psychopathology and personality dysfunction is partially mediated by dissociation. This particular emotional regulation strategy presents a serious obstacle in the functional development of the brain-mind and is considered a primitive defense style related to splitting mechanisms and fragmentation of the self (17).

Substance use disorders (SUD) and depression are two of the most common mental disorders worldwide (18) and are considerably correlated (19, 20). Both diseases represent serious public health problems and cause enormous expenses for the respective health systems of the world. Regarding the influence of emotions in the etiology of addiction and depression, recent advances in Affective Neuroscience (AN) emphasize overlapping dysregulations within the primary emotion systems, particularly within the SEEKING network, which corresponds to the human medial forebrain bundle and the SADNESS or separation distress network, that corresponds to predominantly opioid controlled areas in subcortical and cingulate structures (21–23). The SEEKING system mediates positive feelings of anticipation and curiosity toward the world and corresponds to Berridge's (24) concept of “wanting.” The SADNESS system, which is activated by the loss of a loved object, generates painful feelings of separation distress and loneliness (25). In this context, depression is framed as an evolutionarily conserved mechanism in which the overactive SADNESS system shuts down the acute panic or protest phase of separation distress and triggers a state of despair which is characterized by sustained overactive SADNESS and discontinuation of the SEEKING driven search for a biological reinforcer (26). This mechanism might be mainly promoted by increased dynorphin activity (27, 28). Similarly, addiction is driven by diminished SEEKING resources, either because of sustained artificial over-stimulation through drug consumption or a general hyperactivity of the SADNESS network (21). Therefore, SUD patients would tend to experience similar despair as patients suffering from depression, but chronically self-medicate this painful emotional state through the use of psychoactive substances. The plausibility of this assumption is emphasized by current progress in the neurobiological research underlining the important role of opioid and dopamine systems in the etiology and treatment of both depression and addiction (29–31).

The predominantly subcortical primary emotion networks are assumed as interdependently connected with secondary order processes linked to the basal ganglia and the limbic system and neocortically based tertiary processes: Secondary processes include acquired functions like attachment behavior as well as internalized object relations, while tertiary processes enable complex cognitive operations such as mentalization and identity narratives (32, 33). These higher order processes might be summarized in the term personality organization. Personality organization is defined as psychological functions, which ensure the maintenance of inner equilibrium and relationships to others (34).

One of the most influential constructs of personality organization was developed by Kernberg (35, 36). This model differentiates between neurotic, borderline, and psychotic levels of personality organization, which develop in a complex interaction of temperament and experienced object relations in the early stages of childhood. According to Kernberg, personality organization is mostly reflected through three levels of functioning: (a) Coherence of identity, meaning the stability of differentiated representations of oneself and others; (b) Maturity of defense mechanisms, describing the ability to cope with internal and external conflicts in a functional way, and (c) Ability to test reality, meaning the capability to differentiate between external and internal stimuli (37). Thereby, a constellation of severe identity diffusion, predominance of primitive defenses like splitting and related mechanisms as well as a relatively intact ability to test reality—the so called “borderline organization”—is seen as a typical foundation for many forms of SUD and mood disorders like depression (38–40). These dysfunctional personality patterns are assumed to develop on account of abusive attachment experiences in early childhood (41, 42).

In correspondence to this, addiction is understood as the result of an ultimately futile attempt to chemically seal gaps within personality structure and therefore artificially regulate otherwise unbearable affective states (43). In fact, several empirical studies showed substantial associations between a prevalent borderline personality organization and SUD (44, 45). Interestingly, connections between insecure attachment and symptoms of mood pathology, as well as addiction, can also be found in non-clinical populations (46, 47).

To enhance the understanding of these disorders, this study tries to examine the common underlying structure of both diseases within an AN framework, regarding the influence of childhood trauma. For this purpose, this study applied the structural equation modeling technique, which has the advantage of being able to estimate the relationship and etiological validity of multiple concepts simultaneously. The main purpose of the present study was to investigate the role of primary emotions and personality organization in the relationship between childhood trauma, depressive symptoms, and addictive behaviors. On the basis of previous research, it was hypothesized that primary and higher order processes are correlated, while the expression of both mental properties are influenced by traumatic relationships in childhood. Therefore, the influence of a traumatic environment in childhood was assumed as connected indirectly to the pathogenesis of addictive behaviors and depressive symptoms, since its repercussions are mediated by despair (low SEEKING, high SADNESS) and deficits in personality organization.

**Figure 1:**
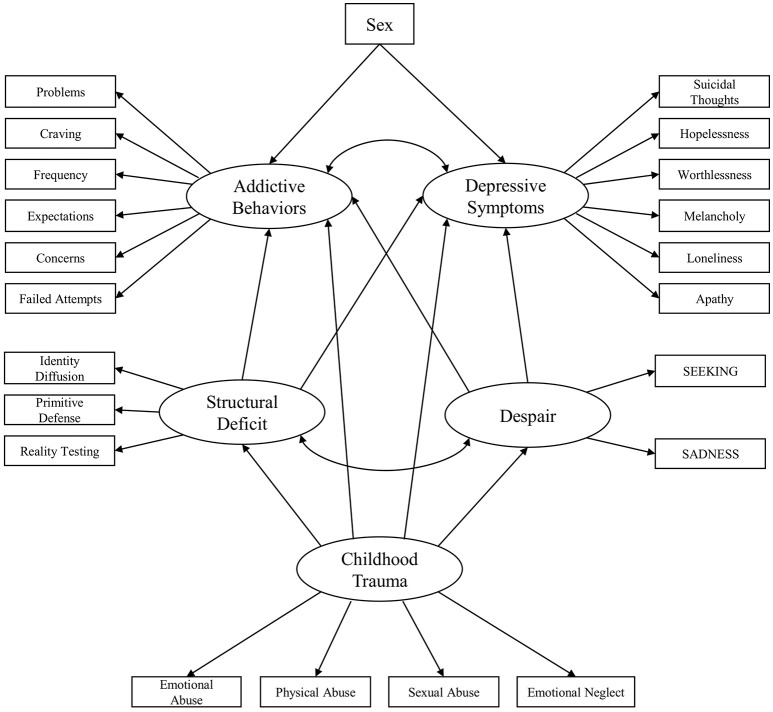
Initial conceptualized framework for the relationship between childhood trauma, despair, personality organization, depression symptoms and addiction symptoms.

Moreover, this study intends to investigate the extent to which addictive and depressive symptoms are predicted by despair, personality organization, sex, and childhood trauma. The path diagram of the conceptual framework corresponding to these hypotheses is presented in Figure 1. Furthermore, epidemiologic literature consistently shows significant sex differences in SUD and depression prevalence, with men being 2 to 3 times more likely to be affected by SUDs (19, 20, 48), while women are 2 to 3 times as likely to experience depression (49–51). Consequently, this study will take the influence of sex into account as a substantial predictor of addictive behaviors and depressive symptoms (Figure 1).

## Materials and methods

### Sample description and procedure

The investigated convenience sample of young adults (52) consisted of 500 German-speaking individuals. The participants were recruited through advertising on social networks such as public forums, Facebook, and public announcements at the University of Graz. Informed consent was acquired before each participant filled in the test form that included demographic questions (e.g., Age, Sex, Education status, and Lifetime psychiatric diagnosis) as well as standardized questionnaires described below. The data was acquired via the online-survey platform LimeSurvey. No recompense was provided. Overall 1502 individuals responded to this online survey. However, 889 discontinued the participation before completion. Participants were included if they were aged between 18 and 39 years, at least graduated high school, stated no recent psychotic episode and completed all questionnaires. On basis of these criteria, 113 participants of the total sample (*n* = 613), who completed the whole survey, were excluded. All participants remained fully anonymous at any time. The study was carried out in accordance with the Declaration of Helsinki. Ethical approval was granted by the Ethics Committee of the Medical University of Graz, Austria. The assessment of participants was carried out from April 2017 to November 2017.

## Psychometric assessment

### Childhood trauma

*The Childhood Trauma Questionnaire* (CTQ) (53), German version by Wingenfeld (54) is a 28-item self-report measure of traumatizing childhood experiences, comprising “Emotional Abuse,” “Physical Abuse,” “Sexual Abuse,” and “Emotional Neglect.” The subscale “Physical Neglect” was excluded in this study due to poor reliability (55). It employs a 1 (“never”) to 5 (“very often”) Likert scale with higher scores indicating more severe abuse or neglect. The subscales showed good to excellent internal consistencies with Cronbach's alpha ranging from 0.83 to 0.93.

### Despair

*The Affective Neuroscience Personality Scale* (ANPS) (56) German version by Reuter and Hennig (unpublished) is a self-report questionnaire which measures behavioral traits related to the concept of subcortical primary emotion circuits, developed by Panksepp (57). Therefore, this questionnaire comprises the subscales “SEEKING,” “SADNESS,” “FEAR,” “RAGE,” “CARE,” and “PLAY” and an additional scale for Spirituality. It consists of, overall, 110 items with 14 items for each subscale and is rated on a 4-point scale ranging from 1 (“strongly disagree”) to 4 (“strongly agree”). Due to theoretical considerations, only the subscales “SEEKING” and “SADNESS” were applied in this study. “SEEKING” summarizes the disposition toward feelings of positive curiosity toward new experiences, the tendency to explore and a sense of being able to achieve relevant goals, while “SADNESS” operationalizes the tendency of feeling separation distress, loneliness and sorrow. Both scales showed acceptable to good internal consistencies, with Cronbach's alpha ranging from 0.78 (SADNESS) to 0.89 (SEEKING).

### Structural deficit

The *16-Item Inventory of Personality Organization* (IPO-16) [German version by Zimmermann et al. (37)], is a self-report measurement of deficits within personality structure. The questionnaire is theoretically grounded in Otto Kernberg‘s (36) model of personality organization. The IPO-16 is comprised of three subscales: (1) “Identity Diffusion,” which measures the integrity of the representations of oneself and others; (2) Dominance of primitive defense mechanisms such as splitting, denial, projection and dissociation (“Primitive Defense”); (3) the capacity to differentiate between internal and external stimuli (“Reality Testing”). A total score of Structural Deficits can be generated. The items are rated on a 5-point Likert scale ranging from 1 (“never”) to 5 (“always”). Internal consistencies for the subscales were acceptable ranging from α = 0.71 to α = 0.81.

### Depressive symptoms

The amount of depressive symptoms was assessed by means of the “Depression” sub-scale of the *Brief Symptom Inventory-18* (BSI-18) (58); German version by Spitzer et al. (59) The BSI-18 is a short version of the Symptom-Checklist SCL-90-R, which assesses the amount of psychological distress in the last seven days and is comprised of 18-items. The total questionnaire consists of the subscales Depression, Anxiety, and Somatization. The items are rated on a 5-point Likert scale ranging from 0 (“absolutely not”) to 4 (“very strong”). Symptoms of depressiveness are assessed by six questions regarding suicidal thoughts, hopelessness, feelings of worthlessness, melancholy, loneliness and apathy. The “Depression” sub-scale showed an excellent internal consistency, with a Cronbach's alpha of 0.91.

### Addictive behaviors

The World Health Organization's *Alcohol, Smoking and Substance Involvement Screening Test* [ASSIST; (60)] is a standardized interview used to detect psychoactive substance use and related problems. For the purpose of this online-study, the ASSIST was adapted as a self-report questionnaire. The questionnaire measures the lifetime use and symptoms of abuse of 10 substance groups including tobacco, alcohol, cannabis, cocaine, amphetamines, inhalants, sedatives, hallucinogens, opioids, and “other drugs.” Symptoms of drug abuse are rated on a 7-point Likert scale from 0 (”never“) to 6 (“daily or almost daily”) for questions 2–5, which assesses the “Frequency of drug use,” “Craving to use the drug,” “Problems” (health, social, legal or financial) because of drug use and “Failed expectations.” Questions 6, 7, and 8 are rated on a 3-point scale (0 = “no, never”; 3 = “yes, but not in the past 3 months”; 6 = “yes, in the past 3 months”) and cover “Expressed concerns by relatives or friends,” “Failed attempts to cut down drug use” and “Drug injection.” The ASSIST can measure several different domains of substance involvement (61). For this study we calculated an overall score for every symptom class related to drug abuse (“Frequency,” “Craving,” “Problems,” “Failed expectations,” “Concerns,” and “Failed attempts to cut down use”) by adding the drug specific symptom scores. We then performed logarithmic transformation of these subscales due to their severe non-normality and implemented the transformed subscales as indicators for the latent variable “addictive behaviors” in the SEM model. The internal consistencies for the subscales implemented as indicators were generally acceptable ranging from α = 0.70 to α = 0.76.

### Statistical analysis and analysis strategy

The measurement model and Structural Equation Modeling (SEM) was conducted with AMOS 18. SPSS 17.0 was used for data management and bivariate correlations. For the model specification, a two-step approach was used (62). The objective of step one was to examine via Confirmatory Factor Analysis (CFA) whether the specified model was a good measurement model. In step two, this specified model was used in the general SEM to estimate path coefficients, indirect effects and mediations.

To establish the goodness-of-fit for the measurement model, a model was developed which loaded theoretically related indicators onto their corresponding latent factors. To assign a metric to the variables, the one path coefficient was constrained to be one for every latent variable. Goodness-of-fit was assessed with a maximum likelihood estimation in AMOS. To test for mediation and indirect effects a bootstrap was performed with a bias-corrected confidence interval of 95% and 1000 bootstrap samples (63).

In accordance with Kline (62), the following fit-indices were considered as markers for an acceptable model fit: (a) The comparative fit index (*CFI*) > 0.90; (b) Tucker Lewis-index (*TLI*) relative fit index > 0.90; (c) The square root error of approximation (*RMSEA*) < 0.08 and the upper bound of its 90% confidence interval < 1. For the comparison of competing models, the Akaike information criterion (*AIC*) was used. The alpha-level was set to 0.01.

## Results

### Sample characteristics and descriptive statistics

The mean age of the participants was 26 years (*SD* = 5.51). 316 (63.2%) were females. 185 (36%) participants declared a university degree as their highest educational level, 199 (39.8%) a general qualification for university entrance, 44 (8.8%) a high school degree, and 72 (14.4%) participants stated a completed apprenticeship as highest educational level. Regarding the current occupation of participants, 163 (32.6%) were in employment, 295 (59.0%) in education, and 42 (8.4%) were unemployed. Concerning the current relationship status, 25 (5%) were married, 227 (45.4%) in a relationship, and 248 (49.6%) were single. The nationality of most participants was either German (*n* = 250; 50%), Austrian (*n* = 202; 40.4%) or Swiss (*n* = 23; 4.6%), while 25 (4.6%) had other nationalities. Finally, 187 (37.4%) participants declared they had been diagnosed with a psychiatric disorder by a psychiatrist. The majority of these participants were diagnosed with depression (*n* = 129; 69%), while 9 (5%) participants stated they had been diagnosed with a form of SUD. Descriptive statistics and zero-order correlations for the variables examined in this study are presented in Table 1.

**Table 1:** Descriptive statistics and zero-order correlations for indicator variables.

**Variable**	**1**	**2**	**3**	**4**	**5**	**6**	**7**	**8**	**9**	**10**	**11**	**12**	**13**	**14**	**15**	**16**	**17**	**18**	**19**	**20**	**21**	**22**
1. ASSIST Frequency	–																					
2. ASSIST Craving	0.70^*^	–																				
3. ASSIST Problems	0.51^*^	0.55^*^	–																			
4. ASSIST Expectations	0.46^*^	0.46^*^	0.57^*^	–																		
5. ASSIST Worries	0.60^*^	0.61^*^	0.53^*^	0.43^*^	–																	
6. ASSIST Failed Attempts	0.56^*^	0.64^*^	0.54^*^	0.43^*^	0.60^*^	–																
7. BSI Apathy	0.23^*^	0.28^*^	0.35^*^	0.28^*^	0.26^*^	0.27^*^	–															
8. BSI Loneliness	0.16*	0.26*	0.30*	0.19*	0.22*	0.25*	0.56*	–														
9. BSI Melancholy	0.16*	0.30*	0.29*	0.23*	0.27*	0.28*	0.59*	0.64*	–													
10. BSI Worthlessness	0.16*	0.28*	0.30*	0.18*	0.23*	0.28*	0.58*	0.71*	0.64*	–												
11. BSI Hopelessness	0.16*	0.27*	0.29*	0.21*	0.21*	0.27*	0.62*	0.67*	0.68*	0.77*	–											
12. BSI Suicidal Thoughts	0.17*	0.22*	0.27*	0.17*	0.22*	0.19*	0.46*	0.56*	0.52*	0.64*	0.60*	–										
13. CTQ Emotional Neglect	0.12	0.19*	0.26*	0.17*	0.23*	0.23*	0.30*	0.35*	0.31*	0.41*	0.36*	0.28*	–									
14. CTQ Sexual Abuse	0.02	0.12	0.09	0.08	0.14	0.13	0.18*	0.15*	0.15*	0.24*	0.19*	0.21*	0.34*	–								
15. CTQ Physical Abuse	0.12	0.13	0.17*	0.09	0.18*	0.20*	0.21*	0.22*	0.19*	0.29*	0.27*	0.21*	0.49*	0.44*	–							
16. CTQ Emotional Abuse	0.16*	0.20*	0.21*	0.13	0.25*	0.22*	0.31*	0.38*	0.34*	0.48*	0.40*	0.32*	0.74*	0.41*	0.60*	–						
17. IPO Identity Diffusion	0.16*	0.24*	0.34*	0.27*	0.18*	0.22*	0.38*	0.48*	0.38*	0.44*	0.40*	0.32*	0.19*	0.16*	0.13	0.29*	–					
18. IPO Primitive Defence	0.33*	0.42*	0.49*	0.35*	0.42*	0.38*	0.41*	0.46*	0.39*	0.45*	0.44*	0.42*	0.31*	0.20*	0.25*	0.39*	0.57*	–				
19. IPO Reality Testing	0.19*	23*	0.33*	0.22*	0.23*	0.18*	0.37*	0.32*	0.26*	0.31*	0.30*	0.35*	0.23*	0.18*	0.20*	0.30*	0.44*	0.56*	–			
20. ANPS SEEKING	−0.01	−0.07	−0.14	−0.13	−0.04	−0.11	−0.40*	−0.26*	−0.27*	−0.31*	−0.36*	−0.20*	−0.24*	−0.11	−0.13	−0.14	−0.18*	−0.16*	−0.07	–		
21. ANPS SADNESS	0.11	0.24*	0.27*	0.16*	0.20*	0.25*	0.46*	0.63*	0.57*	0.62*	0.62*	0.46*	0.34*	0.18*	0.17*	0.40*	0.55*	0.48*	0.29*	−0.31*	–	
22. Sex	−0.29*	−0.19*	−0.15*	−0.13	−0.18*	−0.15*	−0.04	−0.02	0.00	0.07	0.01	0.00	−0.01	0.17*	0.03	0.13	0.05	−0.01	−0.02	0.02	0.14	–
M or N	2.19	2.00	0.81	0.87	1.45	1.27	2.32	2.57	2.39	2.11	2.33	1.57	12.70	6.50	6.69	10.72	15.21	11.52	9.25	2.84	2.67	332
SD or %	0.82	1.12	1.11	1.10	1.21	1.17	1.26	1.40	1.35	1.37	1.43	1.05	5.95	3.52	3.52	5.36	4.62	4.73	4.04	0.40	0.45	61.71

*n = 500*;

* *p < .001; Sex was coded as: 0 = male; 1 = female*.

### Measurement model

The measurement model, which was constructed to test our hypotheses, consisted of five latent variables (Addictive Behaviors, Depressive Symptoms, Childhood Trauma, Despair and Structural Deficit) as well as the single indicator sex.

The following variables loaded onto the latent Addictive Behaviors factor: Frequency (β = 0.78), Craving (β = 0.83), Problems (β = 0.72), Failed Expectations (β = 0.60), Concerns (β = 0.76) and Failed Attempts (to cut down use) (β = 0.75). The following variables loaded onto the latent Depressive Symptoms factor: Suicidal Thoughts (β = 0.69), Hopelessness (β = 0.87), (Feeling of) Worthlessness (β = 0.87), Melancholy (β = 0.77), Loneliness (β = 0.81), and Apathy (β = 0.70). Furthermore, variables loading onto the latent Childhood Trauma factor were: Emotional Abuse (β = 0.94), Physical Abuse (β = 0.66), Sexual Abuse (β = 0.47), and Emotional Neglect (β = 0.77). The following variables loaded onto the latent Despair factor: SADNESS (β = 0.81) and SEEKING (β = −0.37). The following variables loaded onto the latent Structural Deficit factor: Identity Diffusion (β = 0.68), Primitive Defense (β = 0.86) and Reality Testing (β = 0.63). All indicators loaded significantly onto their corresponding latent factors (*p* < 0.001). The specified measurement model exhibited good fit: *RMSEA* = 0.06 (90% *CI*: 0.06, 0.07); *TLI* = 0.92; *CFI* = 0.93; *AIC* = 728.180.

As shown in Table 2 all latent variables showed significant correlations among each other (*p* < 0.001), while female sex was negatively correlated with Addictive Behaviors (*p* < 0.001) and showed a low positive correlation with Despair (*p* < 0.01). The correlations between sex and Childhood Trauma, as well as Structural Deficit exhibited no significance (*p* > 0.01).

**Table 2:** Correlations among latent variables and sex for the measurement model.

**Variable**	**1**	**2**	**3**	**4**	**5**	**6**
1. Addictive Behaviors	–					
2. Depressive Symptoms	0.39^**^	–				
3. Despair	0.33^**^	0.90^**^	–			
4. Structural Deficit	0.56^**^	0.64^**^	0.71^**^	–		
5. Childhood Trauma	0.29^**^	0.52^**^	0.51^**^	0.46^**^	–	
6. Sex	−0.25^**^	0.02	0.16^*^	0.01	0.12	–

*n = 500*;

* *p < 0.01*;

** *p < 0.001; Sex: female = 1; male = 0*.

Due to the remarkably high correlation between Despair and Depressive Symptoms (*r* = 0.90), an alternative model was tested, which nested both latent variables into an overarching factor we termed “Depression.” This alternative measurement model exhibited a good fit as well: *RMSEA* = 0.06 (90% *CI*: 0.06, 0.07); *TLI* = 0.92; *CFI* = 0.93; *AIC* = 740.041. Compared to the initially hypothesized model, the *AIC* of the alternative model was larger with approximately Δ12 (740.041 compared to 728.180), indicating that the initial model was more parsimonious than the alternative nested model and therefore statistically superior.

### Structural equation modeling

**Figure 2:**
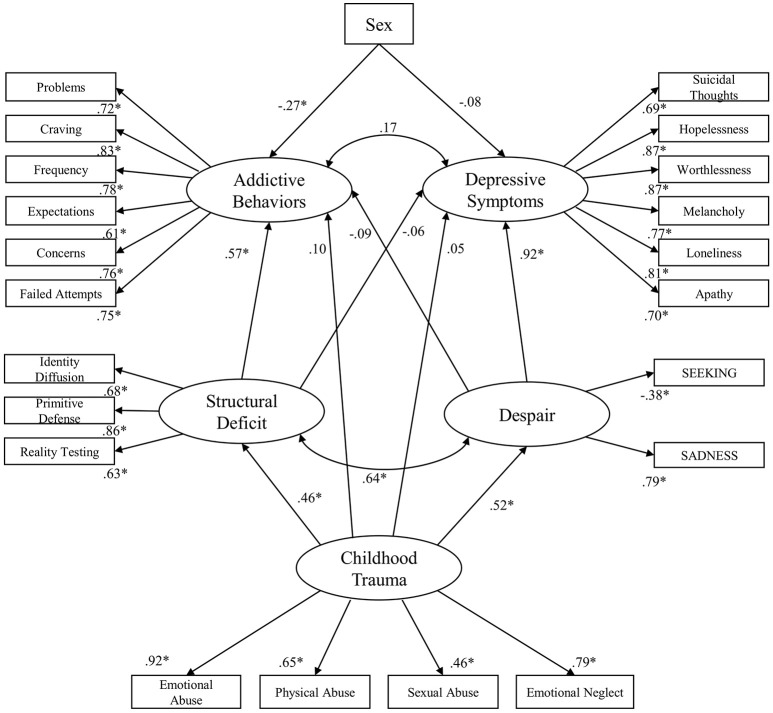
Initial standardized solution for the structural equation model; *p < 0.001; Sex: Female = 1; Male = 0.

The initial standardized solution for the structural equation model is presented in Figure 2. The standardized solution for the structural equation model showed a good fit: *RMSEA* = 0.06 (90% *CI*: 0.06, 0.07); *TLI* = 0.92; *CFI* = 0.93; *AIC* = 737.575. In this model, deficits in Structural Deficit positively predict Addictive Behaviors (β = 0.57; *p* < 0.001) but not Depressive Symptoms (*p* > 0.05) and was significantly correlated with Despair (*r* = 0.64; *p* < 0.001). Furthermore, Despair positively predicts Depressive Symptoms (β = 0.92; *p* < 0.001), while the association between Despair and the Addictive Behaviors was not significant (*p* > 0.05). In addition, the correlation between Depressive Symptoms and Addictive Behaviors was not significant in this model (*p* > 0.05). Moreover, male sex positively predicted Addictive Behaviors (β = −0.27; *p* < 0.001), and showed a weak tendency to predict Depressive Symptoms (β = −0.08; *p* = 0.01). Childhood Trauma significantly predicted deficits in Structural Deficit (β = 0.46; *p* < 0.001) and Despair (β = 0.52; *p* < 0.001), while its association to Addictive Behaviors and Depressive Symptoms exhibited no significance (*p* > 0.05).

However, bootstrap analysis revealed significant indirect effects of Childhood Trauma on Addictive Behaviors (β = 0.22; *p* < 0.01) and Depressive Symptoms (β = 0.44; *p* < 0.01). Its indirect effect on Depressive Symptoms was mediated through its association with Despair, while the indirect effect on Addictive Behaviors was mediated through its association to Structural Deficit.

**Figure 3:**
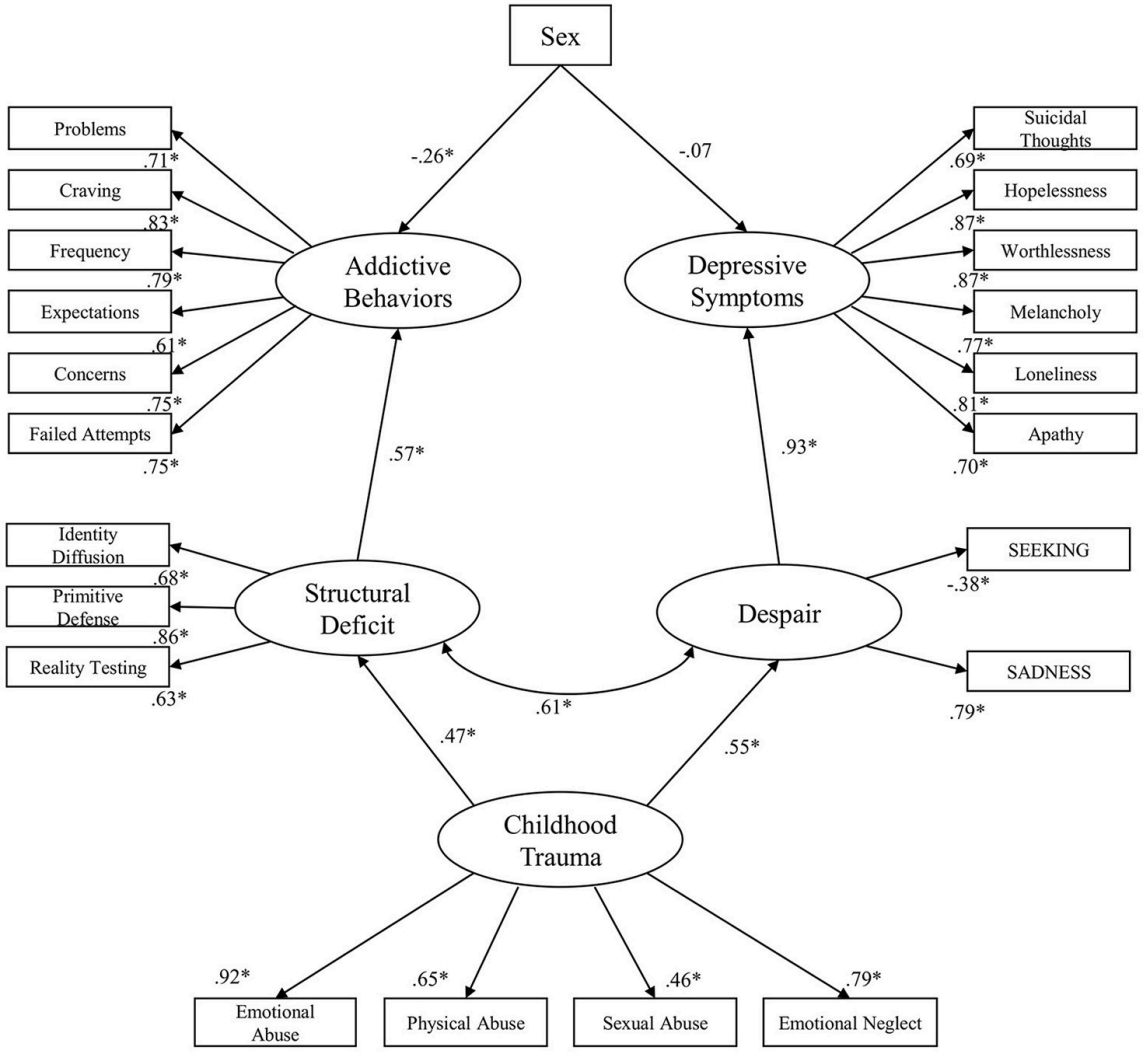
Final standardized solution for the structural equation model; *p < 0.001; Sex: Female = 1; Male = 0.

A further step was utilized as a pruning strategy in which non-significant paths were removed in order to establish the final model. The final standardized solution for the structural equation model is presented in Figure 3. This model showed good fit: *RMSEA* = 0.06 (90% CI: 0.06, 0.07); *TLI* = 0.92; *CFI* = 0.93; *AIC* = 734.855. Then, compared to the initially hypothesized model, the *AIC* of the final model was smaller with approximately Δ3 (737.575 compared to 734.855), indicating that the final model was more parsimonious than the initial model and therefore we observed a better fit for the data. In sum, this model was able to explain 39% of the variance of Addictive Behaviors and 85% of the variance of Depressive Symptoms.

## Discussion

This study examined the role of specific primary emotions and personality organization in explaining the relevance of childhood trauma for the development of depressive symptoms and addictive behaviors in young adulthood. In general, our results suggest that the putative link between childhood trauma and depression is mediated by a primary emotion disposition toward decreased SEEKING and increased SADNESS, while its association with addictive behaviors is mediated by deficits within personality organization. These findings correspond to the increasing amount of literature emphasizing the importance of childhood trauma in the etiology of mental disorders in adults (64). Moreover, the results enhance the understanding of the mechanisms underlying this connection, by disclosing the significant influence of childhood trauma on adult life, primary emotion disposition, and personality organization. The theory that personality organization, in addition to primary emotions, might form on account of early object relations is supported (35, 42). Furthermore, recent studies suggested relevant associations between childhood trauma and impaired white matter integrity, especially in the cingulum, and superior longitudinal fasciculus (65). Therefore, the neurological damage of childhood trauma might be a link to results indicating decreased white matter integrity in the superior longitudinal fasciculus in patients suffering from addiction (66–68), depression (69–71) and deficits in personality organization (66).

Furthermore, the results show a strong correlation between primary emotions and personality organization, which is in line with assumptions of AN and neuropsychoanalytic theory, which assumes an interdependent relationship between primary and higher order processes (33, 42, 72). Therefore, this result emphasizes the important role of defense mechanisms, identity and reality testing in the modulation of primary affective states. Nonetheless, this result might also suggest that primary emotions influence the formation of personality structure. However, this study tested a recursive model, examining only a correlation between primary process emotions and the higher order process concept of personality structure. Therefore, interpretations regarding the direction of influence between these two concepts have to remain speculative at this point. Future studies might investigate more complex models of the affective-cognitive frame and consider non-recursive relationships between primary, secondary and tertiary processes in order to describe this interplay of brain functions in more detail.

Notably, the underlying factors—childhood trauma, primary emotions and personality organization—diminish the correlation between addictive behaviors and depression symptoms when taken into account within a single predictive model. This result is consistent with AN theory, which postulates a common etiological core for addiction and depression (21, 23). However, this study found that decreased SEEKING and increased SADNESS very precisely describe depression symptoms, while their association with addictive behaviors remained repressed by the influence of personality structure. This finding supports the idea that addiction might emerge predominantly as a compensative strategy to seal gaps within a corrosive personality structure (43, 73). In contrast, this result was inverted in the prediction of depression, in which the contribution of personality structure was diminished by the influence of primary emotions. This supports the assumption that depression might be understood as a mechanism that is characterized by a chronically overactive SADNESS system which is linked to reduced activation within the SEEKING system (26). Moreover, this finding is in line with recent research by Montag et al. (74), which was able to demonstrate the great significance of primary emotions in the prediction of depression. Due to these promising results, future studies might try to empirically investigate the AN framework for a broader range of mental disorders.

As a matter of fact, this study observed an associative overlap between despair and depressive symptoms to such an extent, that it was difficult to discriminate between despair as equivalent to symptoms of the disorder and despair as etiologically significant predictor. Therefore, we tested an alternative model, considering the possibility of an overarching “Depression” factor, which however exhibited a higher AIC value than the initial non-nested model and was therefore regarded as statistically inferior to the initially proposed model. Furthermore, on a clinical level it might be more useful for the development of new treatment options for depression, if despair is regarded as an underlying affective mechanism predicting depressive symptom development rather than nesting both constructs.

It should be noted that this study did not explore whether there might be other clinically useful concepts regarding the influence of primary emotions in the development of both disorders. A study by Unterrainer et al. (67), applying variance analysis, was able to observe increased dispositions to SADNESS, RAGE and FEAR in addiction patients as opposed to healthy subjects. Moreover, Montag et al. (75) observed FEAR and SADNESS as predictors for internet addiction. By contrast, in this study the structural equation model was constructed in line with the assumption that addiction and depression might be mediated by a common affective core, consisting of low SEEKING and high SADNESS as proposed in Zellner et al. (21) and Solms et al. (23). While we found significant correlations between despair and addictive symptoms, future studies might deepen exploratory investigations regarding the most predictive constellation of primary emotions for SUDs.

Furthermore, the results of this study indicate male sex as a significant predictor of addiction symptoms, which is in line with recent epidemiological findings (19, 20). However, this study was not able to replicate findings that would suggest female sex as a predictor for depression (49, 50). This quite unexpected finding might be explained due to a self-selection bias within our sample. Male subjects might have been more interested in taking part in this survey if they were affected by signs of this disorder, which might have led to an overrepresentation of male participants exhibiting depressive symptoms.

Some limitations of this study have to be noted. First, the investigated sample in this study contains a relatively high frequency of participants diagnosed with a mental disorder. This might be explained by a self-selection bias in our online recruitment, too. However, we decided not to exclude these participants in order to be able to investigate the continuum between health and pathology. Furthermore, epidemiological studies show that a surprisingly high proportion of one third of the population in a community sample can be expected to be diagnosed with a mental disorder (76), which is close to the 37.4% we observed in our sample. Moreover, lifetime diagnosis was assessed by a question asking, if participants have ever been diagnosed with a psychiatric condition and a follow up question, which asked for the specific diagnosis. In future research the topic of psychiatric disorders might be assessed in greater depth by means of standardized clinical interviews. Notably, the awareness of psychotic symptoms is often hardly present in the concerned individuals. Therefore, we cannot completely rule out the fact, that the investigated sample might include some self-unrecognized cases. This issue could also be addressed in future research by employing enhanced clinical assessment. In addition, the structural equation modeling results imply the possibility of causality, however the design was cross-sectional and therefore associative in nature. Moreover, this study relied on self-report measures that reflect consciously available representations of primary emotion dispositions and personality organization, whereas these concepts are hypothesized to be at least partly unconscious (33, 36). Therefore, replication of these findings with other means of data collection (like qualitative interviews considering less conscious mental states) would strengthen the validity of the results. Finally, a majority of our sample was female which is a common phenomenon in psychological research (77). However, we included sex as a covariate in our model in order to control for possible confounding effects.

## Conclusions

Despite these limitations, the results indicate that the relationships of childhood trauma with primary emotions and personality organization are valid avenues to understanding the emergence of addiction and depression. Thus, these concepts should be regarded in the development and implementation of therapeutic interventions for these disorders. Traumatic childhood experiences are (indirectly) associated with both disorders hence the suggestion that therapeutic discourse concerning biographical material be a part of therapy in general. However, the present results entail that the restructuring of problematic dispositions toward SEEKING and SADNESS may be especially important in the treatment of depression. By contrast, addiction treatment might rather emphasize the development of more mature defense mechanisms, more coherent identity narratives and improved reality perception. Furthermore, future studies on the relationships of childhood trauma with primary emotions, personality organization and psychopathology might also profit from including spirituality as an additional variable. Aspects of spirituality are not only considered as important in addiction treatment (78), they can also be linked to primary emotions as well as personality dimensions (79). In addition, especially existential well-being seems to counteract the influence of insecure attachment on affective symptoms (46).

Lastly, it is important to note that our findings imply an interdependent relationship between primary emotions and personality organization, as well as a significant correlation between depression and addiction. Therefore, both aspects of the affective-cognitive framework could be a useful subject of the therapeutic process in these disorders.

## Availability of data

The raw data supporting the conclusions of this manuscript will be made available by the authors, without undue reservation, to any qualified researcher.

## Ethics statement

This study was carried out in accordance with the recommendations of the ethics guidelines of the Medical University of Graz. The protocol was approved by the ethics committee of the Medical University of Graz. All subjects gave written informed consent in accordance with the Declaration of Helsinki.

## Author contributions

JF and HU conceptualized the study. JF collected, analyzed and interpreted the data. JF, HU and MH-R drafted the manuscript. AK and H-PK critically reviewed the manuscript. All authors gave their final approval of the manuscript.

### Conflict of interest statement

The authors declare that the research was conducted in the absence of any commercial or financial relationships that could be construed as a potential conflict of interest.
